# Endometrial microbiome: sampling, assessment, and possible impact on embryo implantation

**DOI:** 10.1038/s41598-022-12095-7

**Published:** 2022-05-19

**Authors:** Marco Reschini, Laura Benaglia, Ferruccio Ceriotti, Raffaella Borroni, Stefania Ferrari, Marta Castiglioni, Davide Guarneri, Luigi Porcaro, Paola Vigano’, Edgardo Somigliana, Sara Uceda Renteria

**Affiliations:** 1 Infertility Unit, Fondazione IRCCS Ca’ Granda Ospedale Maggiore Policlinico, Via M. Fanti, 6, 20122 Milan, Italy; 2 Dept of Clinical Sciences and Community Health, Università Degli Studi Di Milano, Milan, Italy; 3 Virology Unit, Fondazione IRCCS Ca’ Granda Ospedale Maggiore Policlinico, Milan, Italy; 4 Medical Genetics Laboratory, Fondazione IRCCS Ca’ Granda Ospedale Maggiore Policlinico, Milan, Italy

**Keywords:** Microbiology, Biomarkers

## Abstract

There is growing interest on the potential clinical relevance of the endometrial microbiome. However, insufficient attention has been given to the methodology of sampling. To minimize contamination, we advocate the use of the double-lumen catheters commonly employed for the embryo transfer. Endometrial fluid samples obtained from 53 women scheduled for IVF were studied for microbiome characterization. Control samples from the vagina of these same women were concomitantly obtained. Samples were analysed by V3–V4–V6 regions of 16S rRNA gene sequencing with Next Generation Sequencing technique. Endometrial *Lactobacillus*-dominant cases were uncommon compared to previous evidence, being observed in only 4 (8%) women. Taxonomy markedly differed between the endometrial and vaginal microbiomes composition. The most common bacterial genera coincided in only 4 (8%) women. The comparison between women who did and did not subsequently become pregnant failed to identify any microorganism associated with the success of the procedure. However, the endometrial biodiversity resulted higher among pregnant women. Shannon’s Equitability index in pregnant and non pregnant women was 0.76 [0.57–0.87] and 0.55 [0.51–0.64], respectively (p = 0.002). In conclusion, the use of embryo transfer catheters for testing the endometrial microbiome is promising. The scant concordance with vaginal samples supports the validity of this approach. Moreover, our study highlighted a possible beneficial role of a higher biodiversity on endometrial receptivity.

## Introduction

The reproductive microbiome is an emerging topic in the field of obstetrics and gynecology^1–6^. In particular, some interest is given to the presence of microorganisms within the endometrial cavity, an anatomical niche where low-biomass microorganisms could modulate the local immune environment of the uterus^7^. This may influence the implantation of the embryo and the initial formation of the placenta, potentially affecting fertility and the development of obstetric complications in later phases of gestation^4^. Recent studies suggested that, in an In Vitro Fertilization (IVF) context, a non-Lactobacillus-dominated endometrial microbiota (defined as < 90% Lactobacillus spp.) was associated with significant decrease in implantation, pregnancy and live birth rates^8^.

The sequencing-based methods for bacterial detection are currently the cornerstone of the assessment of the microbiome in low-biomass anatomical sites. These metabarcoding techniques are based on the amplification and sequencing of the bacterial 16S ribosomial RNA (rRNA) gene, a highly conserved gene of bacteria and archaea containing nine hypervariable regions (V1-V9) allowing to discriminate and quantify the different microbial species present in a specific sample^9^. One of the widely used methods for the study of the 16S rRNA region is the Next Generation Sequencing (NGS) where an optimal study designs and faultless methodologies as sample collection and storage, microbial DNA extraction and purification, PCR amplification, sequencing and data cleaning are mandatory to ensure reliability and reproducibility of the results^4^.

The endometrial niche can be easily accessible, and this has boosted the interest on this local microbiome, with some groups hastily claiming for clinical applications^8^. However, insufficient attention has been given to the methodology of sampling. Several and inconsistent procedures have been reported^1^. Of utmost relevance here is the possibility of contamination of the endometrial specimen with cervical or vaginal microorganisms. This concern is fuelled by the knowledge that the vagina and the cervix are high-biomass microbic anatomical districts, with densities of microorganisms that are, respectively, at least 10^7^ and 10^5^-fold higher than those estimated to be present in the endometrial cavity^10^. A minimal contamination with vaginal or cervical secretions can totally subvert the findings. Accordingly, studies investigating the endometrial microbiome using a transcervical catheter for sampling and those using uterine specimen obtained at hysterectomy and reaching the endometrial cavity through transfundal technique showed radically different findings, both in terms of density of microorganisms and colonizing species^1^. The latter approach should undoubtedly viewed as a gold standard but, in vivo, it would expose women to significant and unjustified risks, thus hampering the possibility of clinical applications.

In the present study, we suggest employing the double-sheated catheters commonly used for embryo transfers to obtain endometrial specimens for microbiota assessments. This double-lumen catheter system is expected to markedly shrink the possible contamination with the cervical or vaginal microbiota. To investigate the validity of this modality of sample collection, we compared endometrial and vaginal microbiotas in a series of women undergoing IVF and scheduled for frozen embryo transfer. As a secondary aim, we also evaluated the association of microbiotas’ findings with the subsequent chance of pregnancy.

## Materials and methods

### Study population

Women who were undergoing IVF or IntraCytoplasmic Sperm Injection (ICSI) cycles in the Infertility Unit of the Fondazione IRCCS Ca’ Granda Ospedale Maggiore Policlinico, Milan, Italy between January 2018 to March 2019 were considered for study entry. We exclusively included women who had frozen blastocysts to transfer. Exclusion criteria were as follows: (1) past or current diagnosis of Pelvic Inflammatory Disease (PID), (2) pathological leucorrhea, (3) presence of hydrosalpinx, (4) clinically relevant abnormalities of the endometrial cavity, including fibroids grade 0–3 according to FIGO classification^11^, endometrial polyps and uterine septum, (5) antibiotics assumed during the last month, (6) hormonal treatment (progestins, estroprogestins or gonadotropins) assumed during the last month, (7) vaginal bleeding (for cycling or any other reasons), (8) previous difficult embryo transfers, (9) embryo transfer planned in the same menstrual cycle. Given the importance of the possible confounding effect of recent hormonal or antibiotic use, these items were actively investigated with the woman with specific direct questions and underlining the relevance of this aspect for the success of our research. Both women with regular and irregular menstrual cycles could be included. Women agreeing to participate were informed about the aim of the study, the possible discomfort of the procedure and the possible risks. Only women signing the informed consent could be selected. The study was approved by the local Institutional review Board (Comitato Etico Milano Area B, N.1710/2017) and all research was performed in accordance with the Declaration of Helsinki. For women included in the study, the clinical management was performed according to the routine clinical practice of our Unit^12,13^. No additional tests or attention were given. The full de-anonymized dataset supporting the findings of this study is available upon request to the corresponding author.

### Sample collection

**Figure 1:**
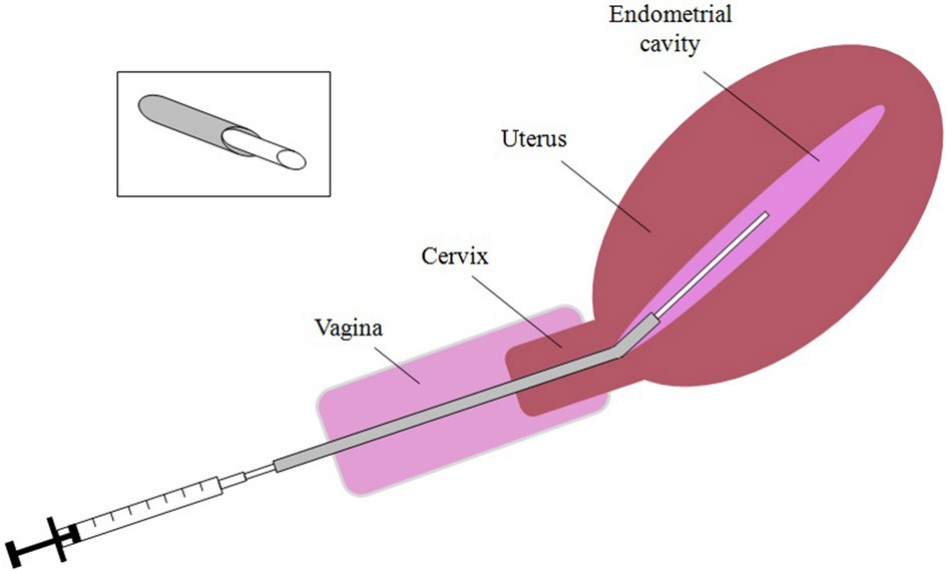
The double-lumen catheter used for endometrial sample collection. This type of catheter is commonly used for embryo transfer. The outer hollow catheter (represented in grey) is placed with its distal part just above the internal os of the cervix. Thereafter, the inner catheter (with a smaller diameter, represented in white) can easily pass through the first one, avoiding contacts with the vaginal and cervical mucosae. Once reached the endometrial cavity, endometrial collection of the endometrial fluid can be obtained by gentle traction on the syringe. The box of upper left corner of the figure shows in more details the mechanism of the double-lumen system.

Eligible women were recruited at the time of the preliminary preparation for the frozen/thawed embryo transfer (all women in our unit undergo a general clinical, sonographic, and biochemical evaluation prior to organize the transfer). The woman was placed in a lithotomic position in a gynecological couch. The sampling was performed as illustrated in Fig. 1. Three healthcare providers were concomitantly involved. A physician placed the devices, a biologist performed the aspiration and handled the specimen, and a nurse assisted the whole process and performed the transabdominal ultrasound to guide the introduction of the first catheter. All of them wore surgical masks. The physician and the biologist also wore sterile gloves. A vaginal speculum was first inserted and the first specimen of vaginal secretion for microbiome assessment was taken from the posterior fornix. To this aim, we used a vaginal sterile swab. Thereafter, repetitive cleaning with abundant sterile saline of the cervix and the vagina was performed. The first outer catheter was then inserted under ultrasound guidance taking much care to avoid any contact with the vaginal walls (it this occurred, the catheter was replaced). Thereafter, we introduced the second inner catheter taking again attention to avoid any contact with nonsterile surfaces. Once the second catheter was inserted in the upper part of the endometrial cavity, the biologist performed a firm aspiration with a 20 ml syringe while the catheter was slowly retrieved within the endometrial cavity. A large syringe was chosen to perform a steady and strong negative pressure that could allow effective aspiration of the scant endometrial fluid. Then, aspiration was stopped, the second catheter was removed, and the minimal content was gently suspended in the 150 μl of sterile saline previously prepared in a 1 ml sterile Eppendorf tube. The distal part of the catheter (2–3 mm) was then cut with sterile scissors and let inside the Eppendorf tube together with the saline. The Eppendorf tube was then closed and stored at − 80 °C together with the vaginal swab until assessed. Evaluation of the samples were grouped and done some months after the sampling. Therefore, all the personnel who managed the patients was blinded to the results. Once results were available, women found to have bacterial vaginosis were contacted to perform a vaginal culture and, if positive, they were treated.

### Bacterial DNA extraction and NGS Sequencing

At the time of assessment, samples were thawed and bacterial DNA was isolated using QIAamp DNA Microbiome kit (Qiagen, Hilden, Germany), following manufacturer’s indications. It was reported that QIAamp method is efficient in the host DNA contamination depletion and microbial DNA enrichment^14^. The extracted DNA was used for the library preparation and assessment of multiplex real-time PCR for detection of bacterial vaginosis.

NGS of the bacterial 16SrRNA gene was performed by PCR amplifying, library preparation and sequencing using microbiota solution B kit for hypervariable regions V3–V4–V6 (Arrow Diagnostics srl, Italy) as reported in detail elsewhere^15^. Briefly, PCR amplification of the hypervariable regions V3–V4–V6 was obtained by employing degenerated primers that lead to the identification of most of the bacterial populations present in the microbiota. Each library was quantified with Qubit 2.0 fluorometer using Qubit dsDNA HS (High Sensitivity) Assay kit (Thermo Fisher Scientific, MA, USA). The sequencing was performed on Illumina MiSeq system platform with MiSeq Reagent Kit v3 (Illumina Inc., CA, USA).

Raw sequencing data were analyzed automatically with MicrobAT software (Microbiota analysis Tool, SmartSeq srl, Italy) providing a report for each sample with Operational Taxonomic Units (OTUs) assignment. Sphingomonas and Arthrobacter were excluded because considered a priori contaminated genera. This assumption was made based on the available literature^3,15^ and confirmed based on the results emerging from some preliminary analyses in our laboratory aimed at identifying possible contaminants using blank control device not in contact with the biological material (such as the tip of the catheter used for the collection of the endometrial sample).

Given the previously reported similarities between the vaginal and the endometrial microbiomes, samples were also tested using Allplex™ Bacterial Vaginosis *plus* Assay (Seegene, Seoul, Korea), a commercially available multiplex real-time PCR kit for quantitative and qualitative detection of bacterial vaginosis related bacteria. This test allows simultaneous amplification and detection of target nucleics acids of *Gardnerella vaginalis*, *Atopobium vaginae*, *Megasphaera* type 1, *Bacterial vaginosis associated bacteria 2*, *Mobiluncus* spp, *Bacteroides fragilis* and *Lactobacillus* spp (*Lactobacillus crispatus, Lactobacillus gasseri, Lactobacillus jensenii)*. The assay provides an automatic Bacterial Vaginosis interpretation as Normal, Intermediate, and Positive using quantitative analysis.

### Statistical analysis

Data was analyzed using the software Statistical Package for Social Sciences (SPSS 23.0, IL, USA). Data was reported as number (%), mean ± SD or median [Interquartile range—IQR]. Comparisons were made using Student’s *t* test or non-parametric Mann–Whitney test or Chi square test or Fisher Exact test, as appropriate. P values below 0.05 were considered statistically significant. The relative proportion of the different bacterial genera was calculated using as a denominator only the total number of informative reads: the rate of non-informative reads was reported for each sample but then excluded for this specific evaluation. Based on previous evidence, endometrial microbiome was considered *Lactobacillus*-dominant if the relative abundance of *Lactobacillus* exceeded 90%^8^. The Shannon index (S_H_) and Shannon’s Equitability Index was calculated by MicrobAT software.

Given the exploratory approach of the study, a robust sample size calculation could not be done. Therefore, we arbitrarily scheduled the number of cases to be recruited at about 50. This sample size would allow us at least to confirm the findings of Moreno et al. who tested the capacity of the endometrial microbiome to predict pregnancy in 35 women (even if this was the secondary aim of our investigation). More specifically, if findings of Moreno et al.^8^ could be replicated with our approach, we should expect 50% of women with normal endometrial microbiome (*Lactobacillus*-dominant). On this basis, setting the type I and II errors at 0.05 and 0.20, and expecting a general chance of pregnancy of 30% for the whole cohort, we would be able to demonstrate statistically significant differences in clinical pregnancy rates in the *Lactobacillus*-dominant and in the non *Lactobacillus*-dominant women > 50% and < 15%, respectively.

### Ethics approval

The study was approved by the local ethical committee (*Comitato Etico Milano Area 2, N. 457/2017*).

### Consent to participate

Patients provided a written informed consent to participate and those denying this consent were excluded.

## Results

A total of 59 women were initially selected. Microbiota could not be completely assessed in four subjects: specifically for two vaginal samples and two both vaginal and endometrial samples. In addition, two women did not perform the embryo transfer (one did never refer for the transfer and one did not have any viable blastocysts after thawing). Data were thus complete and presented for the remaining 53 subjects. Sampling was performed in the proliferative and secretory phases in 26 (49%) and 24 (45%) women, respectively. The remaining three subjects underwent sampling in amenorrhea for disovulatory disorders. Baseline characteristics of women who did and did not become pregnant after frozen transfers are shown in Table 1.

**Table 1:** Baseline characteritics and cycle outcome of the studied population and of pregnant and non-pregnant women.

Characteristics	All subjects (n = 53)	IVF Outcome		
		Not pregnant (N = 37)	Pregnant^a^ (N = 16)	p
Age (years)	34 [32–37]	35 [32–37]	34 [33–37]	0.94
BMI (Kg/m^2^)	22.0 [19.9–24.2]	22.0 [19.8–24.3]	22.0 [19.8–23.0]	0.70
Current Smokers	10 (19%)	9 (24%)	1 (6%)	0.25
AMH (ng/ml)	3.34 [2.23–4.65]	3.50 [2.60–4.70]	2.80 [1.75–5.19]	0.54
AFC	12 [8–17]	12 [8–16]	14 [8–22]	0.33
Previous intrauterine pregnancies	37 (70%)	26 (70%)	11 (69%)	1.00
Previous live births	24 (45%)	18 (49%)	6 (38%)	0.55
Duration of infertility (years)	3 [1–4]	3 [1–4]	3 [2–4]	0.33
**Cause of infertility**				0.36
Male factor	19 (36%)	12 (32%)	7 (45%)	
Tubal factor	5 (9%)	3 (8%)	2 (12%)	
Endometriosis	7 (13%)	5 (14%)	2 (12%)	
Unexplained infertility	19 (36%)	16 (43%)	3 (19%)	
Mixed	3 (6%)	1 (3%)	2 (12%)	
**Total N. of previous oocytes retrievals**				0.31
1	37 (70%)	27 (73%)	10 (62%)	
2	11 (21%)	8 (22%)	3 (19%)	
≥ 3	5 (9%)	2 (5%)	3 (19%)	
**Endometrial preparation for transfer**				1.00
HRT	14 (26%)	10 (27%)	4 (25%)	
Natural cycle	39 (74%)	27 (73%)	12 (75%)	
**Menstrual phase at time of sampling**				0.22
Proliferative	26 (49%)	21 (57%)	5 (31%)	
Secretory	24 (45%)	14 (38%)	10 (63%)	
Amenorrhea	3 (6%)	2 (5%)	1 (6%)	

AFC was obtained in the early menstrual phase at the time of the ovarian hyperstimulation cycle. AMH was assessed regardless of the menstrual phase. Data is reported as number (%), mean ± SD or Median [interquartile range], as appropriate.

*N* Number, *BMI* Body Mass Index, *AFC* Antral Follicle Count, *AMH* Anti-Mullerian Hormone, *HRT* Hormone Replacement Therapy, *IQR* Interquartile Range.

^a^All women underwent elective single embryo transfer. Pregnancies that could be obtained with the transfer of additional remaining blastocysts were excluded.

The median [IQR] total number of sequence reads for endometrial and vaginal samples were 2,583 [385–6,083] and 13,822 [7,310–26,920] respectively. The median [IQR] Shannon indexes of the endometrial and vaginal samples were 2.83 [2.49–3.17] and 1.70 [1.44–2.22] (p < 0.001), respectively. The median [IQR] Shannon’s Equitability indexes of endometrial and vaginal samples were 0.57 [0.52–0.74] and 0.44 [0.40–0.53] (p < 0.001), respectively. Precise results of bacterial abundance at genus level for the endometrial and vaginal samples are shown in Supplemental Table 1 and Supplemental Table 2, respectively. The most relevant findings are summarized in Table 2. Overall, Lactobacillus prevailed in 16 (30%) and 29 (55%) endometrial and vaginal samples, respectively. Fourteen (88%) of the 16 women who had *Lactobacillus* as most common specie in the endometrial samples also had *Lactobacillus* as most common specie in the vaginal samples. Samples could be classified as *Lactobacillus*-dominant (abundance of *Lactobacillus* > 90%) in 4 endometrial (8%) and 22 vaginal (42%) specimen, respectively. The Spearman correlation index between the proportion of *Lactobacillus* in the endometrial and vaginal samples was modest (Rho = 0.53, p < 0.001). The dominant bacterial genera of the endometrial and vaginal microbiota coincided in only 4 (8%) women. For endometrial microbiota, the most found bacterial genera were *Lactobacillus, Pelomonas, Probionabacterium, Pseudomonas, Streptococcus and Escherichia shigella*. For the vaginal microbiota, they were *Lactobacillus*, *Gardnerella* and *Bifidobacterium*. When considering separately subjects in the proliferative and secretory phases, no main differences emerged (detailed data not shown). In particular, the Lactobacillus relative abundance in the endometrium did not differ, being 15.0% [2.5–44.5%] and 7.0% [2.5–37.5%], respectively (p = 0.61).

**Table 2:** Abundance of the most prevalent bacteria in endometrial and vaginal microbiota.

Site	Bacterial genus	Samples with prevalence > 1%	Distribution of the prevalence^a^ (%)	Most prevalent species	Dominant species (> 90%)
Endometrial cavity	*Lactobacillus*	44 (83%)	13 [3—37]	16 (30%)	4 (8%)
	*Propionibacterium*	42 (79%)	7 [4–14]	2 (4%)	0 (0%)
	*Pelomonas*	39 (74%)	6 [2–9]	4 (8%)	0 (0%)
	*Pseudomonas*	32 (60%)	8 [2–14]	3 (6%)	0 (0%)
	*Streptococcus*	30 (57%)	2 [1–3]	1 (2%)	0 (0%)
	*Escherichia shigella*	27 (51%)	4 [1–11]	4 (8%)	2 (4%)
Vagina	*Lactobacillus*	48 (91%)	83 [27—99]	29 (55%)	22 (42%)
	*Gardnerella*	17 (32%)	31 [15—62]	8 (15%)	1 (2%)
	*Bifidobacterium*	14 (26%)	50 [8—87]	7 (13%)	4 (8%)

For the most common bacteria, the table illustrates the frequency of specimen clearly demonstrating its presence (prevalence > 1%), their Median [interquartile range] distribution, when they are the prevalent species (“Most prevalent species”) and the frequency of specimen showing a strong dominance of that species (“Dominant species”, i.e., when the species represented > 90% of the detected bacteria). Data is reported as number (%) or Median [interquartile range], as appropriate.

^a^This data refers exclusively to samples showing a prevalence of the genera above 1%.

**Figure 2:**
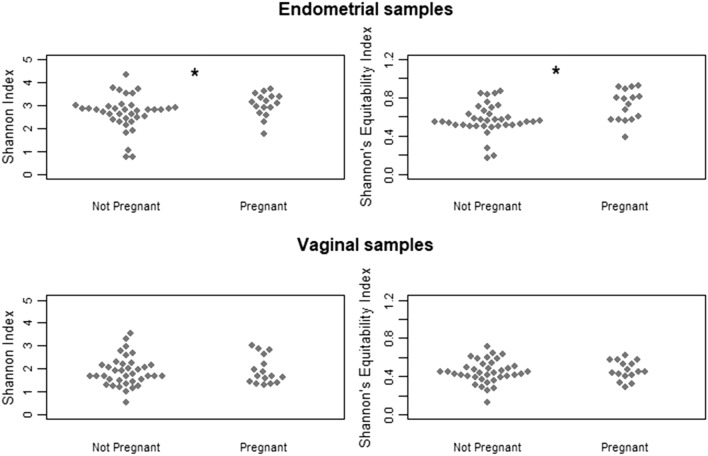
Shannon and Shannon’s equitability indexes in women who did and did not become pregnant. A statistically significant difference emerged for both indexes in the endometrial microbiomes (p = 0.036 and p = 0.002, respectively). Conversely, no significant differences could be observed for the vaginal microbiomes.

Sixteen women became pregnant (30%), ten (19%) had a live birth (these cases are highlighted in green in the Supplemental Tables). In the 16 pregnant women, the number of cases where *Lactobacillus* prevailed in the endometrial and vaginal samples was 5 (29%) and 9 (53%), respectively (p = 0.16). For live birth, these numbers were 3 (33%) and 4 (40%) (p = 0.64), respectively. Two of the 4 subjects classified as *Lactobacillus* dominant in endometrial sample became pregnant. Results of presence and distribution of bacterial species in the vaginal and endometrial samples in subjects who did and did not become pregnant are illustrated in Supplemental Figs. 1 and 2. No significant differences could be observed. Shannon index for endometrial samples between pregnant and not pregnant women was 3.11 [2.72–3.38] and 2.80 [2.40–2.95] (p = 0.036), respectively. Shannon’s Equitability index in pregnant and not pregnant women was 0.76 [0.57–0.87] and 0.55 [0.51–0.64] (p = 0.002), respectively (Fig. 2, upper panel) suggesting a higher biodiversity in association with pregnancy establishment. Figure 2 (bottom panel) shows Shannon and Shannon’s Equitability indexes in vaginal sample, but no significant differences could be detected between pregnant and non-pregnant women.

Data from the multiplex real-time PCR for bacterial vaginosis in the endometrial and vaginal specimens are also presented in detail in Supplemental Table 1 and Supplemental Table 2, respectively. Overall, bacterial vaginosis could be detected in four vaginal samples and in none of the endometrial samples. Three of these four women with vaginal bacterial vaginosis achieved pregnancy.

## Discussion

Results from the use of the careful and meticulous modality of endometrial sampling reported in the present study are comforting. The observation that taxonomy between the endometrial and vaginal microbiomes composition markedly differed supports the validity of our sampling method. A strong overlap between the two would have not allowed to discern whether the two niches were colonized by the same species or, conversely, whether the endometrial samples were contaminated by vaginal species. Moreover, as secondary findings, we failed to confirm the previously reported beneficial effect of *Lactobacillus*-dominant endometrial microbiota on the success of IVF, neither a detrimental effect of bacterial vaginosis as assessed with commercially available multiplex real-time PCR. Interestingly, we observed higher Shannon and Shannon’s equitability indexes among women who became pregnant.

The vagina and the cervix are high-biomass districts and significant contamination can occur by just transiting through the cervix. Of utmost relevance here is that the density of microorganisms in the cervix was estimated to be 10^5^-fold higher compared to the endometrial cavity^10^. Our sampling method aimed at overcoming the limitations of the commonly used transcervical collection^1^. In contrast to previous findings, microbiome composition of endometrium and vagina frequently differed in our data. In fact, the similarity between the two could not be used to question the modality of sampling (the districts are contiguous, and one could speculate that the endometrial microbiomes may be strongly influenced by the vaginal and cervical ones), but, in our opinion, the observation of marked differences is a strong point in favor of the accuracy of our technique of sampling.

Our modality may overcome contamination even if, obviously, it cannot be considered ideal. One may argue that the first catheter may be contaminated after the insertion and that the passage of the second catheter through the first one to reach the endometrial cavity may also contaminate our specimens. On the other hand, one has to recognize that further improvement of the technique is difficult to foresee. Trans-myometrial sampling would obviously overcome this minimal exposure but, from a clinical perspective, this would require a trans-abdominal approach that is invasive, potentially risky and also exposed to the risk of contamination with the cutaneous microbiome.

The use of the catheters for embryo transfer to assess the microbiome is not entirely new. The technique was used by one of the first groups who investigated the endometrial microbiome^16,17^. However, these authors used catheters that were just employed for the transfer of the embryos. As a matter of fact, the authors could not perform the active aspiration that was applied in our protocol, contamination could have occurred in the phases of embryo charging/transport and findings could have been influenced by the recent oocyte retrieval (in particular in case of antibiotics use and fresh transfer). More recently, Liu et al. also used a double-sheathed catheter to obtain an endometrial sample, thus a very similar technique to ours^18^. Surprisingly, the presence of *Lactobacillus* in endometrial samples was higher compared to our findings, the median relative abundance being 81%. Explanations for this inconsistency are difficult to disentangle. On the other hand, it has to be underlined that Carosso et al. who also relied on the use of double-lumen catheters for embryo transfer showed a mean rate of *Lactobacillus* of 27%^15^, more in line with our findings. To note, Qiu et al. who employed a hysteroscopic-assisted collection of specimens with the aim of circumvent contaminations, reported an even lower frequency of *Lactobacillus* of 7%^19^. This latter methodology is however more complicated and invasive than the one herein proposed. Interestingly*,* Verstraelen et al. who used a transcervical sheathed brush device (consisting in a brush protected by a sheath up to the uterine cavity) and conceived to diagnosis endometrial cancer, also failed to show Latobacillus dominance in endometrial microbiome^20^. This device is expected to have the same effectiveness in protecting the specimens from cervical contamination than the double-lumen catheter that we used in our study. However, it is rougher for the integrity of the endometrium.

Our study also provided evidence on the impact of the endometrial microbiome on the chances of pregnancy with IVF. However, our findings are in contrast with studies performing simple transcervical sampling^8^. Of utmost relevance is the scant frequency of lactobacillus-dominant cases in our series and the absence of any relation of this genera with pregnancy rate. To note, our findings do not really question the findings of Moreno et al. (i.e., a remarkable difference in the frequency of lactobacillus-dominant women among those who became pregnant), but question the fact that these authors really had data on endometrial microbiome. It seems more plausible that their inferences were done on the cervical microbiome. From a clinical perspective, this may actually be unremarkable.

An unexpected but intriguing result of our study is the relation between higher alpha-diversity and the chance of pregnancy. The two measures of within subjects diversity (Shannon and equitability indexes) highlighted statistically significant differences between women who did and did not get pregnant. It is tempting to speculate that, in the endometrial microbiome, a higher biodiversity rather than a lactobacillus-dominant milieu is beneficial to pregnancy. Further studies are warranted to confirm and investigate more in depth this fascinating aspect. To note, this evidence comes from exploratory evaluations (it was not the primary aim of the study) and, as such, should be interpreted with caution.

Finally, data from the use of the multiplex real-time PCR for bacterial vaginosis indicate that this method is not adequate for the analysis of endometrial specimens. Indeed, bacterial vaginosis was never detected in endometrial samples. If, on one hand, we failed to setup a simplified and already validated mode of microbiome testing, on the other hand our data confirm that the two compartments, the vaginal and the endometrial, have different colonizing microorganisms.

Some strengths and limitations of the study need to be discussed. Even if the sample size is large for an exploratory investigation and allowed to replicate the findings from Moreno et al., it is insufficient to rule out a milder but potentially still clinically relevant predictive capacity of the endometrial microbiome. Secondly, a referral standard is lacking. Comparisons of our findings with microbiome data obtained from hysterectomy specimen of the same patients would be a more informative design. However, such study design has also some weaknesses. Only older women with gynecologic diseases can be recruited and, in addition, performing the endometrial samples with our technique prior to hysterectomy may theoretically contaminate the endometrial cavity, thus altering the results of the subsequent transmyometrial sampling after the removal of the uterus. Thirdly, we speculate that the microbiome of the vagina and the cervix overlap. This may not be entirely true. Lastly, we did not attempt to collect samples exclusively in the secretory phase, when embryo implantation generally takes place. Menstrual phase could modify local microbiome^21^.

In conclusion, our data support the use of embryo transfer catheters associated to a scrupulous aseptic methodology for testing the endometrial microbiome. To note, a shared and pragmatic modality for sampling is fundamental for future basic and clinical studies. We even plea for an international consensus on this issue and, in this context, we believe that our technique should receive utmost consideration. Moreover, our study highlighted a possible beneficial role of a higher biodiversity on endometrial receptivity. This result is intriguing and appealing but emerged from secondary exploratory analyses and deserves therefore validation in future studies.

## Supplementary Information

Supplementary Information 1.

Supplementary Information 2.

Supplementary Information 3.

Supplementary Information 4.

Supplementary Information 5.

## Data Availability

The datasets used and/or analysed during the current study available from the corresponding author on reasonable request.

## References

[1] Benner M, Ferwerda G, Joosten I, van der Molen RG (2018). How uterine microbiota might be responsible for a receptive, fertile endometrium. Hum. Reprod. Update..

[2] Agostinis C, Mangogna A, Bossi F, Ricci G, Kishore U, Bulla R (2019). Uterine immunity and microbiota: A shifting paradigm. Front. Immunol..

[3] Hashimoto T, Kyono K (2019). Does dysbiotic endometrium affect blastocyst implantation in IVF patients?. J. Assist. Reprod. Genet..

[4] O'Callaghan JL, Turner R, Dekker Nitert M, Barrett HL, Clifton V, Pelzer ES (2020). Re-assessing microbiomes in the low-biomass reproductive niche. BJOG.

[5] Altmäe S, Rienzi L (2021). Endometrial microbiome: New hope, or hype?. Reprod. Biomed. Online..

[6] Molina NM, Sola-Leyva A, Haahr T, Aghajanova L, Laudanski P, Castilla JA, Altmäe S (2021). Analysing endometrial microbiome: Methodological considerations and recommendations for good practice. Hum. Reprod..

[7] Al-Nasiry S, Ambrosino E, Schlaepfer M, Morré SA, Wieten L, Voncken JW, Spinelli M, Mueller M, Kramer BW (2020). The interplay between reproductive tract microbiota and immunological system in human reproduction. Front. Immunol..

[8] Moreno I, Codoñer FM, Vilella F (2016). Evidence that the endometrial microbiota has an effect on implantation success or failure. Am. J. Obstet. Gynecol..

[9] Chakravorty S, Helb D, Burday M, Connell N, Alland D (2007). A detailed analysis of 16S ribosomal RNA gene segments for the diagnosis of pathogenic bacteria. J. Microbiol. Methods..

[10] Chen C, Song X, Wei W, Zhong H, Dai J, Lan Z, Li F, Yu X, Feng Q, Wang Z, Xie H, Chen X, Zeng C, Wen B, Zeng L, Du H, Tang H, Xu C, Xia Y, Xia H, Yang H, Wang J, Wang J, Madsen L, Brix S, Kristiansen K, Xu X, Li J, Wu R, Jia H (2017). The microbiota continuum along the female reproductive tract and its relation to uterine-related diseases. Nat. Commun..

[11] Munro MG, Critchley HO, Broder MS, Fraser IS (2011). FIGO working group on menstrual disorders. FIGO classification system (PALM-COEIN) for causes of abnormal uterine bleeding in nongravid women of reproductive age. Int. J. Gynaecol. Obstet..

[12] Cardellicchio L, Reschini M, Paffoni A, Guarneri C, Restelli L, Somigliana E, Vegetti W (2017). Frozen-thawed blastocyst transfer in natural cycle: Feasibility in everyday clinical practice. Arch. Gynecol. Obstet..

[13] Benaglia L, Busnelli A, Biancardi R, Vegetti W, Reschini M, Vercellini P, Somigliana E (2018). Oocyte retrieval difficulties in women with ovarian endometriomas. Reprod. Biomed. Online..

[14] Heravi FS, Zakrzewski M, Vickery K, Hu H (2020). Host DNA depletion efficiency of microbiome DNA enrichment methods in infected tissue samples. J. Microbiol. Methods..

[15] Carosso A, Revelli A, Gennarelli G, Canosa S, Cosma S, Borella F, Tancredi A, Paschero C, Boatti L, Zanotto E, Sidoti F, Bottino P, Costa C, Cavallo R, Benedetto C (2020). Controlled ovarian stimulation and progesterone supplementation affect vaginal and endometrial microbiota in IVF cycles: A pilot study. J. Assist. Reprod. Genet..

[16] Franasiak JM, Werner MD, Juneau CR, Tao X, Landis J, Zhan Y, Treff NR, Scott RT (2016). Endometrial microbiome at the time of embryo transfer: Next-generation sequencing of the 16S ribosomal subunit. J. Assist. Reprod. Genet..

[17] Tao X, Franasiak JM, Zhan Y, Scott RT, Rajchel J, Bedard J, Newby R, Scott RT, Treff NR, Chu T (2017). Characterizing the endometrial microbiome by analyzing the ultra-low bacteria from embryo transfer catheter tips in IVF cycles: Next generation sequencing (NGS) analysis of the 16S ribosomal gene. Hum. Microbiome J..

[18] Liu Y, Ko EY, Wong KK, Chen X, Cheung WC, Law TS, Chung JP, Tsui SK, Li TC, Chim SS (2019). Endometrial microbiota in infertile women with and without chronic endometritis as diagnosed using a quantitative and reference range-based method. Fertil. Steril..

[19] Qiu T, Liu L, Zhou H, Sheng H, He Y, Liu M, Cai H (2021). Analysis of endometrial microbiota in intrauterine adhesion by high-throughput sequencing. Ann. Transl. Med..

[20] Verstraelen H, Vilchez-Vargas R, Desimpel F, Jauregui R, Vankeirsbilck N, Weyers S, Verhelst R, De Sutter P, Pieper DH, Van De Wiele T (2016). Characterisation of the human uterine microbiome in non-pregnant women through deep sequencing of the V1–2 region of the 16S rRNA gene. PeerJ.

[21] Sola-Leyva A, Andrés-León E, Molina NM, Terron-Camero LC, Plaza-Díaz J, Sáez-Lara MJ, Gonzalvo MC, Sánchez R, Ruíz S, Martínez L, Altmäe S (2021). Mapping the entire functionally active endometrial microbiota. Hum. Reprod..

