# Supplementary figures and images for: Endometrial microbiome: sampling, assessment, and possible impact on embryo implantation
Source: Sci Rep. 2022 May 19;12:8467. doi: 10.1038/s41598-022-12095-7 (PMC9120179; doi:10.1038/s41598-022-12095-7)

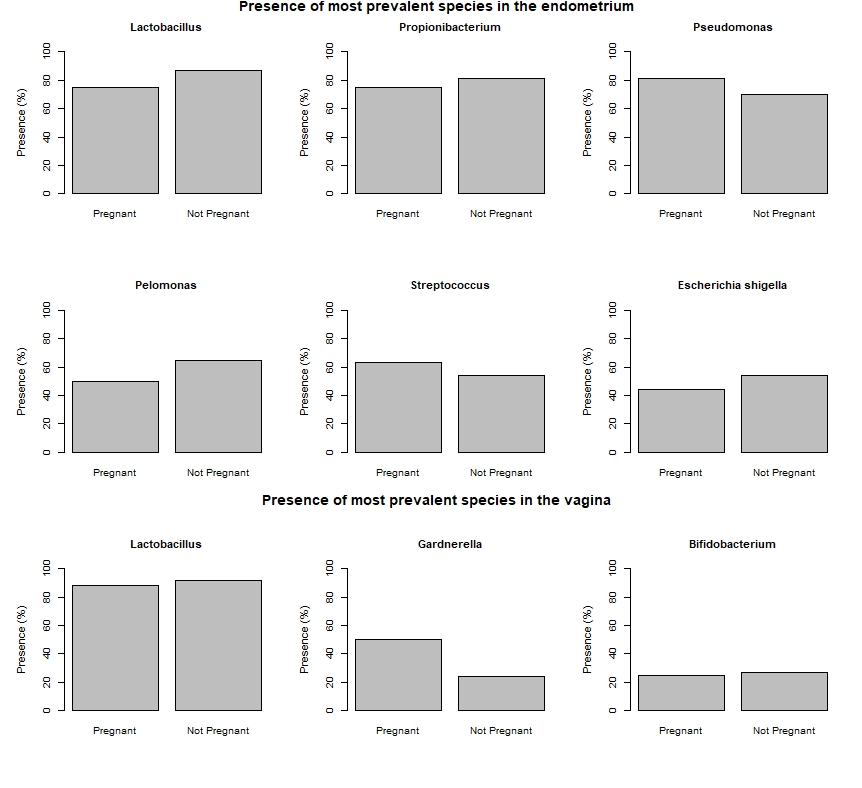

Supplement: Supplementary file 1 — Supplementary Information 1. [file 41598_2022_12095_MOESM1_ESM.jpg]

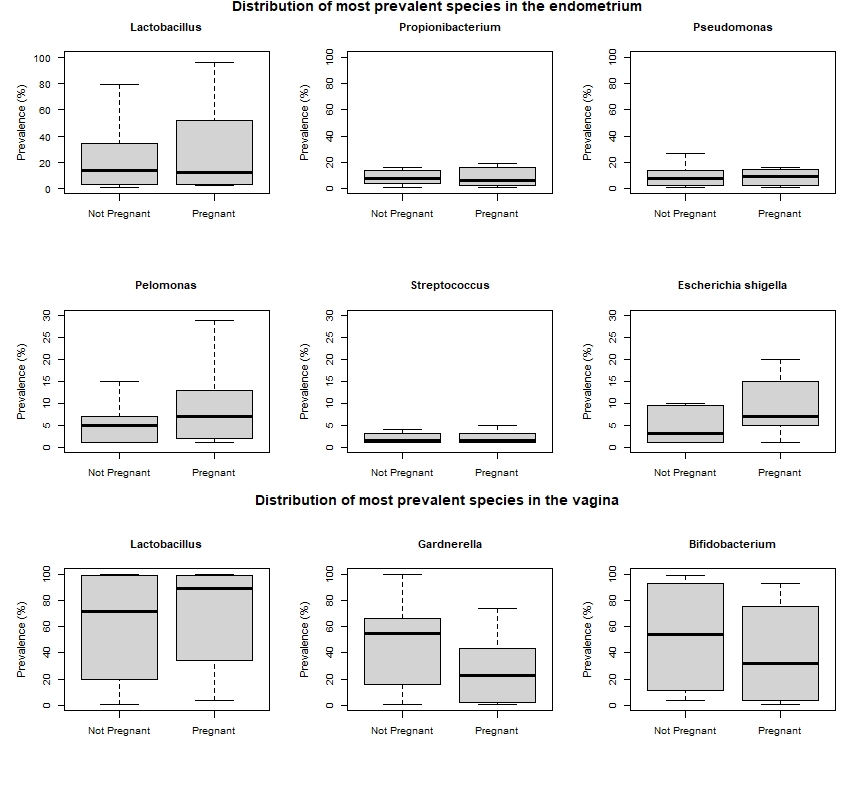

Supplement: Supplementary file 2 — Supplementary Information 2. [file 41598_2022_12095_MOESM2_ESM.jpg]
