# Supplementary material for: Endometrial microbiome: sampling, assessment, and possible impact on embryo implantation
Source: Sci Rep. 2022 May 19;12:8467. doi: 10.1038/s41598-022-12095-7 (PMC9120179; doi:10.1038/s41598-022-12095-7)
Supplement: Supplementary file 5 — Supplementary Information 5. [file 41598_2022_12095_MOESM5_ESM.docx]

**Supplemental Figure 1:** Presence of the most common bacterial species in the endometrial (upper part) and vaginal (lower part) samples in women who did and did not become pregnant. No significant differences could be observed.

**Supplemental Figure 2:** Distribution (relative proportion) of the most prevalent bacteria species in the endometrial (upper part) and vaginal (lower part) samples in women who did and did not become pregnant. No significant differences could be observed.
